# A Bibliometric Analysis of Research on Supported Ionic Liquid Membranes during the 1995–2015 Period: Study of the Main Applications and Trending Topics

**DOI:** 10.3390/membranes7040063

**Published:** 2017-11-07

**Authors:** Ricardo Abejón, Heriberto Pérez-Acebo, Aurora Garea

**Affiliations:** 1 Chemical and Biomolecular Engineering Department, University of Cantabria, Avda. Los Castros s/n, 39005 Santander, Spain; aurora.garea@unican.es; 2 Mechanical Engineering Department, University of the Basque Country UPV/EHU, P° Rafael Moreno “Pitxitxi” 2, 48013 Bilbao, Spain; heriberto.perez@ehu.eus

**Keywords:** supported ionic liquid membranes, separation, bibliometric analysis, research trends

## Abstract

A bibliometric analysis based on Scopus database was performed to identify the global research trends related to Supported Ionic Liquid Membranes (SILMs) during the time period from 1995 to 2015. This work tries to improve the understanding of the most relevant research topics and applications. The results from the analysis reveal that only after 2005 the research efforts focused on SILMs became significant, since the references found before that year are scarce. The most important research works on the four main application groups for SILMs defined in this work (carbon dioxide separation, other gas phase separations, pervaporation and liquid phase separations) were summarized in this paper. Carbon dioxide separation appeared as the application that has received by far the most attention according to the research trends during the analysed period. Comments about other significant applications that are gaining attention, such as the employment of SILMs in analytical tasks or their consideration for the production of fuel cells, have been included.

## 1. Introduction

Supported liquid membranes (SLMs) must be considered as an effective method for the selective separation of different compounds in a variety of processes. In SLMs, a selected solvent is immobilized into the pores of an inert solid membrane support just by capillary forces [1]. The transport through SLMs is based on liquid-liquid extraction and the mass transfer process can be explained by the solution-diffusion mechanism. The most important step of the separation involves the solute diffusion through the solvent in the pores of the membrane due to a chemical gradient as driving force [2]. The SLM acts as a selective permeable barrier between the feed and receiving phases. This selectivity results from the differences in the membrane permeability to the different chemicals due to their solubility in the solvent. Systems based on SLMs put forward numerous process advantages such as low capital investment and operating cost, low energy consumption, very compact design, low solvent requirement and simple operation [3]. The main drawback of SLMs is their stability, which causes a poor long-term performance characterized by reduced selectivity. The loss of the solvent in the pores of the support is the main reason for this detrimental performance. This loss occurs because of the evaporation of the solvent or its dissolution/dispersion into the adjacent phases [4]. Moreover, some solvents included in SLMs should be avoided due to their toxicity.

**Figure 1:**
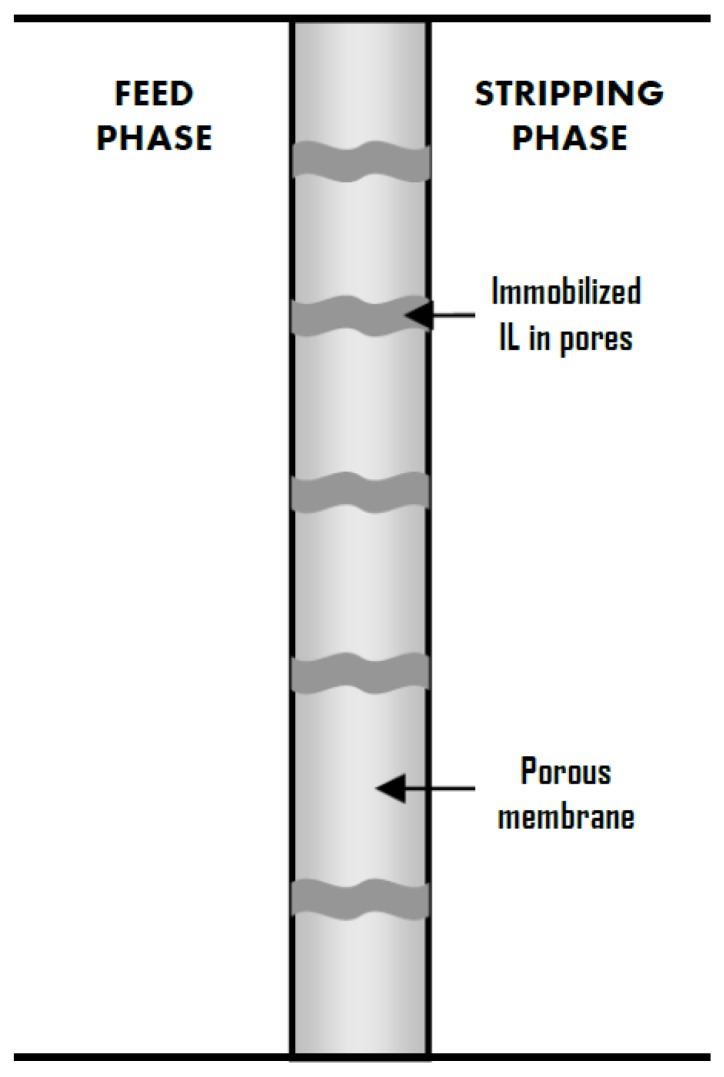
Schematic representation of a SILM (Supported Ionic Liquid Membrane).

Therefore, alternative greener solvents must be preferred and ionic liquids (ILs) can be considered as a very promising option. An IL is a salt in which the ions are poorly coordinated, which results in these solvents being liquid below 100 °C, or even at room temperature. At least one ion has a delocalized charge and one component is organic, which prevents the formation of a stable crystal lattice. Each different ion pair combination results in a different set of physicochemical properties, hence they are considered as “designer solvents” [5]. Consequently, ILs can be formulated to be task specific and therefore, can be tailor made. Nevertheless, some characteristics that are common to most ILs must be highlighted. In particular, the negligible saturated vapour pressure of ILs is very relevant, as the loss due to evaporation is drastically avoided. Besides, their good thermal stability and non-flammability can be very useful in the applications that occur at sufficiently high temperatures [6]. Other interesting properties of ILs are their high ion conductivity [7] and high solvent power [8]. In contrast to common organic solvents, these intrinsic properties of ILs make them ideal liquid phases for SLMs applications. This way, supported ionic liquid membranes (SILMs) can be obtained, which can be defined as SILMs in which the traditional solvent has been replaced by a given IL (Figure 1). SILMs can overcome the stability problems of SLMs, since not only the displacement of the liquid phase from the membrane pores through solvent evaporation is minimized but also the use of ILs allow the formation of more stable systems thanks to their higher intrinsic viscosity and surface tension [9]. In addition, the employment of SILMs allows the minimization of the amount of IL required for a given process, with the corresponding reduced economic costs and greatly facilitates the recovery and reusability of the IL [10]. Thin membranes with short diffusion paths are preferred to be employed for SILMs because they can compensate for the slow mass transfer due to the high viscosity of ILs. The proper selection of the most convenient IL for each SILM application is crucial, as high mutual solubility of water or other solvents in many ILs will restrict their use in those situations [11].

In the last decade, much research effort has been expended on reporting the employment of SILMs for the separation of different compounds—organic compounds such as amines, alcohols, organic acids, ketones, ethers and aromatic hydrocarbons, gas mixtures or metal ions. Some review papers have been focused on the development and application of SILMs [12,13,14,15]. These documents can provide an overview of the recent advances in SILMs, including issues regarding methods of preparation, transport mechanisms, configurations, stability and fields of application. However, bibliometric studies that analyse this topic have not been identified.

A bibliometric analysis is a very valuable tool to obtain good information and knowledge about the status of scientific research activities in specific disciplines, which help researchers to recognize novel trends and interests within investigation frameworks [16]. This technique utilizes statistical indices and quantitative analyses to assess the contributions of authors, institutions and countries in terms of their research output but it can be also useful in identifying relevant aspects related to qualitative features of scientific research output [17]. These bibliometric analyses were developed in the 1960s and can be applied to evaluate comprehensively the large number of documents that are being published [18]. Academic publications in scientific journals are considered the most important resources for this type of bibliometric analysis [19]. Bibliometric methods have been used to measure scientific progress in many disciplines of science and engineering and are a common research instrument for systematic analysis of research trends [20,21,22,23,24,25,26,27,28,29,30,31,32].

The main aim of this work is the bibliometric analysis of the literature published in Scopus-listed publications from 1995 to 2015 related to the research on SILMs. The systematic evaluation of the documents found was employed to determine the quantitative characteristics of the research and provide an overview of trends in this topic, mainly regarding the most important applications, which, to the best of our knowledge, has not been subject of similar studies previously. Therefore, this paper hopes to be useful to help researchers to understand the global panorama of the research on SILMs and identify the most relevant directions of future research in this area.

## 2. Data Sources and Methodology

The search of available scientific literature was based on the online version of Scopus. This abstract and indexing database with full-text links, which was launched in 2004, is produced by Elsevier and claims to index over 21,500 active titles from more than 5000 international publishers, stating that it is the “largest abstract and citation database of peer-reviewed literature” [33]. An independent and expert advisory board selects the list of indexed titles using strict criteria based on user demand and market research. It contains more than 38 million abstracts with references back to 1996 and more than 22 million records before that year. Around 60% of the covered titles are from countries other than Unites States, highlighting European and Asian literature in English and other languages. Therefore, Scopus offers the broadest, most integrated coverage of peer-reviewed literature across the sciences, technology, engineering and medicine (STEM), as well as social sciences and arts and humanities. In order to improve search recall, in addition to the keywords added by authors, extra index terms are manually included for most of the titles in the database, derived from thesauri that Elsevier owns or licenses.

The online search within Scopus was carried out in March 2017 by the selection of “supported ionic liquid membrane” as keywords in the Article Title, Abstract, Keywords field of the search-engine in order to obtain the complete bibliography with all the articles related to the research on SILMs published during the period from 1995 to 2015. The keywords were introduced together between quotations to select only papers that include those words in that order. Otherwise, any document mentioning one of those words would be selected. The total number of articles found was 187. Some additional searches were tested to have more information about the relevance of SILMs. On the one hand, the search with “ionic liquid” as keywords in the Article Title, Abstract, Keywords field during the same period (1995–2015) resulted in 43,767 documents. On the other hand, an equivalent search with “supported liquid membrane” as keywords found 1549 papers. Therefore, although SILMs represent only a small portion of all the research related to ILs, this topic is quite relevant within SLMs, as it represents above 12% of the total production. Moreover, an increasing importance in the last years must be considered: when the figures for 2014 (100 documents for SLMs and 34 for SILMs) or 2015 are compared (87 and 20 documents, respectively), the relative contributions of SILMs to SLMs show higher values (34% for 2014 and 23% for 2015). 

The research papers found in the online search were analysed to provide a basis for a better understanding of the global research scenario, which could help with the establishment of future long-term strategies within this field. Therefore, the analysed aspects covered not only the quantitative description of the publications (annual outputs, leading countries and institutions, or main journals, languages and Scopus subjects) but also the research trends derived from the analysis of the provided keywords.

## 3. Results and Discussion

### 3.1. Bibliometric Analysis of Research on Supported Ionic Liquid Membranes (1995–2015)

#### 3.1.1. Publication Year, Document Type and Language of Documents

**Figure 2:**
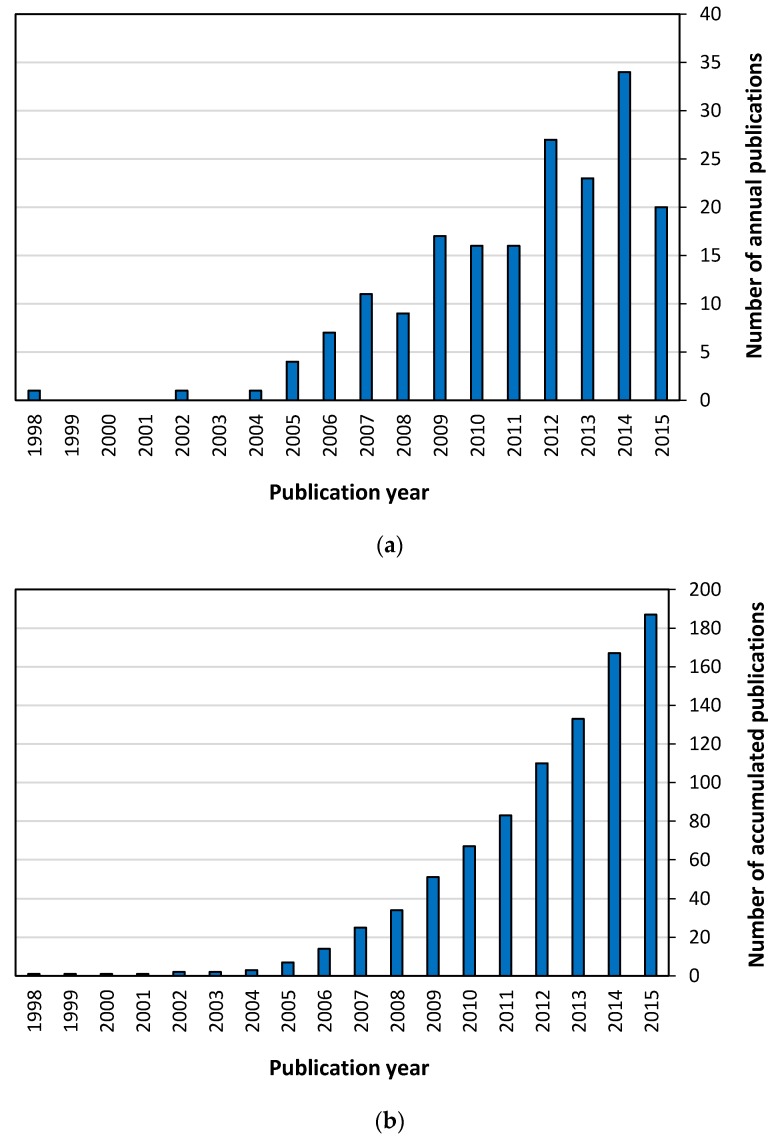
(**a**) Annual and (**b**) accumulated publication output.

The distribution of annual publications and the evolution of the number of accumulated documents are depicted in Figure 2. First of all, it must be remarked that the earliest document found applying the selected criteria was published in 1998. No publications appeared during the 1995–1997 period but even when the search criteria were modified to eliminate the time restriction, the search did not find any additional publication before 1998. Consequently, it was concluded that Tae H. Cho, Joan Fuller and Richard T. Carlin should be considered as the pioneers that treated this topic for first time thanks to their article entitled “Catalytic hydrogenation using supported ionic liquid membranes” [34]. Besides, the production until 2005 was very limited: only other 2 documents were found, which were published in 2002 and 2004.

The graphs in Figure 2 revealed that the publication rate followed a very irregular evolution. While a linear increase could be identified from the data in the 2005–2007 range, this trend was clearly broken. The production in 2008 was lower than in 2007 and the production during the period 2009–2011 was quite constant. Moreover, the production after that year presented a visibly unevenness with twists and turns, which alternated high and low production peaks.

Other databases, such as Web of Science (WoS) and Google Scholar, were also employed to verify these figures. The Web of Science is a scientific citation indexing service maintained by Thomson Reuters. They state that the Web of Science is the leading source of content and tools for ranking organizations around the world [35]. After carrying out the same search, 119 articles fulfilled the requirements. Google Scholar database reported a list of 1060 results in the same period of time. In this case, any item including the reference “supported ionic liquid membranes” appears but any type of selection was performed. Nevertheless, databases as Scopus and WoS only index high quality peer reviewed journals, books and proceedings [33,35] and, hence, the scientific contribution of indexed documents is assured.

The distribution of document types was analysed. Six different document types were found among the 187 publications from the defined time period. Article (137) was the most frequently identified document type comprising 73.3% of the publications, followed by conference paper, with 29 documents (15.5% of the total production). The other less significant categories, which joint contribution was lower than 12%, included review (9), book chapter (9), conference review (2) and book (1). The percentages obtained were concordant with the figures reported by other bibliometric studies that revealed the clear supremacy of articles over other types of publication when analysing the trends on the research about different topics in subjects directly related to chemical or environmental engineering [36,37].

English was clearly the most common language in the documents found (92.5% of the publications were written in English). Only other four languages were found, appearing Chinese as the second language but with a very low contribution, as the papers written in this language only represented 6.0%. The rest of languages were considered anecdotal, since only a document written in Spanish, German and Polish were found. Once again, a bibliometric analysis demonstrated that English is the main language employed for scientific research and more specifically in the engineering field [38,39].

#### 3.1.2. Distribution of Output in Subject Categories and Journals

The distribution of research subjects can be observed in Table 1, where the 6 most popular categories are shown. The categories are non-exclusive and a publication can be related to more than one research subject due to interdisciplinary research. The ranking indicated that *Chemical Engineering* was the dominant category, with a contribution percentage of 72.2%. However, *Chemistry* was a very relevant category too, since 66.8% of the documents fell in this research subject. Therefore, a great collaboration between chemical engineers and chemists should be expected in the topics related to SILMs. *Materials Science* and *Engineering* occupied the 3rd and 4th positions respectively, while *Biochemistry*, *Genetics and Molecular Biology*, in the 5th position, also contributed more than 20%. Out of these categories, none of the other subjects reached the 20% contribution threshold. 

The distribution of publications in journals is shown in Table 2. The corresponding values (year 2015) of Impact Factors (IF) of the Web of Science database and the SCImago Journal Rank (SJR) index of the Scopus database of the top 5 journals (the only ones with more than 5 publications) were also included. The journal that published more documents was *Journal of Membrane Science*, as 35 documents were published in this journal, which represented 20.8% of the total amount of papers. *Separation and Purification Technology* and *Desalination*, two journals highly related to separation processes, completed the podium of the top journals, while two more generalist journals in the field of Chemical Engineering, such as *Chemical Engineering Journal* and *Industrial and Engineering Chemistry Research* completed the list. The high quality of the listed journals (all of them with IF values above 2.5 and *Journal of Membrane Science* and *Chemical Engineering Journal* above 5.3) must be highlighted. Therefore, the research on SILMs must be considered as a very relevant issue within the global investigation scenario regarding chemical engineering.

#### 3.1.3. Publication Distribution of Countries and Institutions

The analysis of author’s countries was based on papers in which the address and affiliation of at least one author was provided. There were 2 papers without any author name, so the total number of documents considered for the analysis of country contribution was 185. Among all the documents with author address, 154 (83.2%) were single country publications and 31 (16.7%) were international collaborative publications. Table 3 shows the top 10 countries ranked by the number of total publications with other information: the ranking and percentage of contributions according to single country or internationally collaborated papers, as well as first authors and corresponding authors. In some categories like total publications or international collaboration rank, since the country affiliation is not exclusive category (a document can be contributed by authors from more than one country), some documents may be classified in more than one country simultaneously due to international collaborations. Therefore, the sum of the number of documents in these categories is above the total number of documents and a similar result can be found when percentages are analysed, with results above 100%. Nevertheless, the contribution percentages of the top 10 countries summed up more than 93% of the total number of documents, fact that gave an idea about the existence of a reduced number of countries that dominate the publication of documents in this research field, in a similar way to other topics in chemical and environmental engineering [40,41]. The analysis demonstrated that USA was the most productive country, with 42 documents, which implies a percentage of 22.7%. This leader country was followed by China (33 documents and 17.8%). These two countries can be found among the top contributors in most scientific fields, including the full list of engineering branches [42,43,44]. Japan is the fourth country in the ranking (16 documents and 8.6%) and it can be also considered as a great contributor to scientific literature [45]. The third position was occupied by Spain (26 documents and 14.1%) and the fifth position was shared by Portugal and Germany (14 documents each and 7.6%). Another two European countries appeared among the top 10 countries, since Czech Republic and Poland shared the 7th position with India. In the 10th position appears United Kingdom. 

The top 8 institutions, the only ones which produced more than 5 documents, were ranked by their number of publications (Table 4). Among these institutions, 3 were Spanish, 2 American and one each from Japan, Portugal and Czech Republic. All these 5 countries appeared among the top 10 most productive countries. However, China, which was the second most productive country, had not any institution situated among the most productive ones, so it can be suspected that the Chinese production was highly shared among several institutions. The leading organization was the Polytechnic University of Cartagena with 14 documents, followed by the National Energy Technology Laboratory (Morgantown) with 13 documents and Doshisha University with 11 documents. Another two Spanish institutions were then found—the University of Murcia and the University of Cantabria. In view of these results, the significant role of Spanish research in the field of SILMs must be highlighted, with 3 universities among the top 5 most productive institutions.

#### 3.1.4. Most Frequently Cited Papers

The 10 most cited papers among the found documents are compiled in Table 5. The range of cites varied from 128 for the paper in the 10th position to 409 for the leading article. Although a further comment on the current research trends will be made in the next section, the analysis of the most cited publications has been useful to identify some important topics that attract attention from main researcher groups investigating SILMs over the world.

After analysis of the most cited papers, the application of SILMs to gas separations and more specifically carbon dioxide separation, must be highlighted, as 8 papers in Table 5 are related to this topic. Among these 8 documents, 4 of them are directly related to carbon dioxide separation and capture, including the most cited one [46,47,48,49].

The other 2 papers in the list of the most cited documents covered different topics. The first one, located in the 5th position, introduced an innovative application of SILMs to analytical purposes [50]. It explained the effectiveness of a SILM to extract chlorophenols from environmental water samples and concentrate them before the corresponding analysis by high-performance liquid chromatography (HPLC). The second one, located in the 8th position, was a very complete review about SILMs, which was prepared by researchers from the Polytechnic University of Cartagena (leading institution in Table 4) with collaboration of researchers from the National Center for Metallurgical Research, which belongs to the Spanish National Research Council [14]. This review covered the main aspects of the SILMs, from the preparation methods or the stability and transport mechanisms to the most important configurations and applications.

### 3.2. Analysis of Author Keywords and Hot Topics of the Research on Supported Ionic Liquid Membranes

Author keywords point out the major attention of the research presented in an article. Therefore, the analysis of the most frequently used author keywords can provide relevant information to identify the research trends in a certain field. Bibliometric studies have considered author keywords analysis in a specific period as a helpful method to find research hotspots [51] and this work followed this approach.

**Figure 3:**
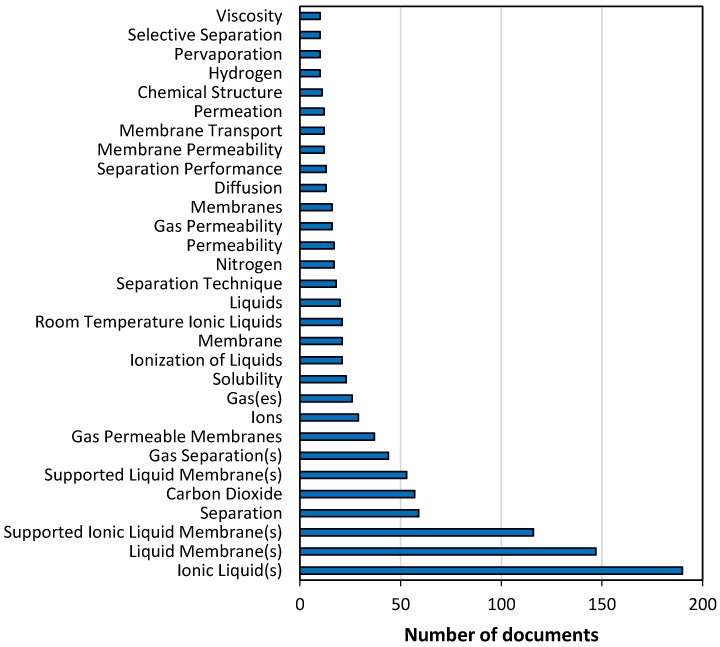
Most frequently used keywords.

The most frequently used keywords, which were mentioned by, at least, 10 of the 187 articles found, are listed in Figure 3.

Singular and plural forms were considered together to simplify the breakdown. The analysis revealed that 30 words or expressions were used as keywords at least 10 times. “Ionic liquid(s)” was the most relevant keyword because the plural form appeared in 121 papers (64.7% of the total amount of papers), while the singular form in 69. It is clear that some documents have selected simultaneously both the singular and plural forms, as the total count supposed 190 documents, more than the number of identified documents (187 papers). The 2nd and 3rd places in the ranking belonged to “Liquid membrane(s)” (147 times) and “Supported ionic liquid membrane(s)” (116 times), respectively. This last expression in its singular form was the exact phrase selected to be introduced in the article title, abstract, keywords field of the search-engine.

Nevertheless, other keywords that were not included in the exact phrase employed for the search can reveal more information about the research trends in this field. Expressions like “Separation” (in the 4th position, employed 59 times) and “Carbon dioxide” (ranked in 5th position with 57 documents) clearly confirmed the impression pointed out from the analysis of the most cited documents: gas phase separation and, specifically, carbon dioxide separation, is the most investigated application of SILMs. Other keywords in Figure 3 corroborated this idea, such as “Gas permeable membranes”, “Gas separation(s)”, “Gas(es)”, “Nitrogen” or “Hydrogen”. On the other side, none of the keywords appearing in Figure 3 but “Liquids” in the 15th position, could be directly associated to liquid phase separations. Keywords related to liquid phase separations were not selected by more than 5 documents. “Water”, with 5 documents and “Aqueous solution”, “Liquid phase” or “Wastewater treatment”, all of them with 4 documents, can be cited as examples. However, the keyword “Pervaporation” did appear among the most mentioned ones in Figure 3. Although the mass transport through a pervaporative membrane occurs in the vapour phase, pervaporation can be considered a separation method for mixtures of liquids by partial vaporization. Nevertheless, other relevant keywords provide information about the main characteristics of the SILMs to be studied. The keyword “Permeability”, which was employed 17 times and some related expressions, such as “Gas permeability” and “Membrane permeability” (selected by 16 and 12 documents, respectively), appeared among the most important keywords. Another crucial characteristic of a SILM is its selectivity and “Selective separation” is compiled in Figure 3 but it is only used in 10 documents. Regarding to the characteristics of the ILs, “Solubility” and “Viscosity” are included as well in the keyword list, so these aspects have been worth attention by researchers that have investigated SILMs.

### 3.3. Review of the Main Applications of Supported Ionic Liquid Membranes and Current Trending Topics

Although the list of potential applications for SILMs is vast, all the applications can be included in one of the following categories:

- Carbon dioxide separation
- Other gas separations
- Pervaporation
- Separations in liquid phase
- Other applications

**Figure 4:**
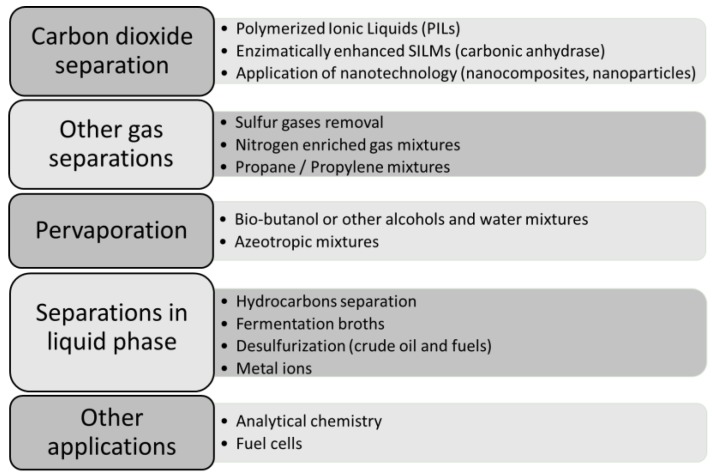
Main applications and hot current trends for SILMs.

A schematic summary about the most important topics investigated by the found references in each category is shown in Figure 4 and a more detailed analysis of each category is carried out in the next subsections on the basis of the most significant documents.

Nevertheless, other relevant topics not directly related to specific SILMs applications, which can be considered common to most SILMs, have been deeply investigated and should be mentioned. For instance, on the one hand, the characterization of SILMs by very different techniques remains as a very hot topic, since these techniques can provide information for a better understanding of the mechanisms involved in the selective transport of SILMs. This is basic to optimize their applicability and implementation in real-scale industrial processes [52,53,54,55]. On the other hand, the analysis of the stability of the SILMs has been object of intensive research efforts, since the underlying liquid phase loss mechanism of SILMs must be taken into consideration in order to minimize it and extend the effective lifetime of SILMs [56,57,58,59,60]. In addition, the importance of imidazolium based ILs must be cited, as they are the most frequently used ILs in the analysed research papers, in agreement with other review documents that have highlighted the relevance of these ILs in SILMs [15,61]. Regarding the membranes, PVDF, PP and PTFE were found to be the most frequently appearing polymeric supports for SILMs. 

#### 3.3.1. Carbon Dioxide Separation

The separation of CO_2_ from industrial gas mixtures for posterior capture or reuse has attracted worldwide interest due to global warming. The strong interaction between the quadrupole moment of CO_2_ and the electrical charge of ILs provides an enhancement in its solubility over other gases, which has converted this field in the most important application for SILMs [15,49]. The efforts in the employment of ILs for CO_2_ separation and the understanding of their properties with respect to CO_2_ have been multiplied during the last decade, as well as the design of new polymers and composite materials to support these ILs in order to develop CO_2_-selective membranes [62,63]. Since very complete review papers that deeply analyse this topic can be found in bibliography [1,64], this subsection will be a very brief approach to the use of SILMs for CO_2_ separation from other gases, with special emphasis on the most recent trends, which are not so extensively covered yet by the available reviews.

The nearly infinite combinations of cations and anions allow the design of ILs with specific properties for the development of SILMs with very high CO_2_ selectivity [65,66]. Some reported SILMs show better separation performance than current commercial materials, such as cellulose acetate. This exceptional separation performance can be evaluated through the analysis of the large number of Robeson plots published. Moreover, the evolution from SILMs to more complex configurations such as gelled ionic liquid membranes (GILMs) or polymer inclusion membranes with ionic liquids (PIMILs) provides a perspective on the continuous evolution of this field [1].

Regarding the analysis of the hottest topics, nanomaterials have emerged as a very promising tool to attain unusual properties that are out of reach for conventional materials [67]. Nanotechnology allows the integration of these nanomaterials into larger components and systems, keeping the control of these new and improved materials at the nanoscale [68]. The application of nanomaterials to CO_2_ separation has been a very relevant topic, including the interactions between nanomaterials and ILs. Some important studies directly related to the integration of nanomaterials and SILMs are reported. For instance, nanocomposite membranes obtained by the “brick and mortar” method were investigated as support for SILMs applied to CO_2_ separation from N_2_ [69]. Carbon black nanoparticle “bricks” were dispersed in soft-templated mesoporous carbon “mortar” to produce nanoporous membranes compatible with [BMIM][NTf_2_] and [EMIM][NTf_2_]. The nanostructure of the membranes stabilized the IL in the pores by providing strong capillary forces, which allows the operation under high transmembrane pressure (around 10 bar, a value that is not possible using conventional polymer and ceramic supports) without degrading their separation performance—CO_2_ permeability up to 180 Barrer with values of CO_2_/N_2_ selectivity around 36. Other recent examples of SILMs that employ nanoparticle-based structures can be found. Kamiya et al. investigated the separation of CO_2_ from air by means of SILMs improved by the presence of nanoparticles [70]. Several ILs were mixed with triethylenetetramine and diglycolamine. Alumina nanoparticles (40–50 nm) were added to this mixture and the resulting fluid was supported in microporous PVDF membranes. The results demonstrated extended effective lifetime of the SILM. Using a similar method, ZnO nanoparticles were dispersed in [BMIM][BF_4_] and supported in PES microporous membranes [71]. The selectivity for CO_2_/N_2_ and the CO_2_ permeance of the SILM were significantly enhanced to 42 and 101, respectively, compared to the neat membrane without nanoparticles, which had CO_2_/N_2_ selectivity and CO_2_ permeance values of 5 and 17. This improvement was justified by the improved CO_2_ solubility due to the presence of the oxide layer. This approach, which proposes the use of nanoparticles as CO_2_ carriers to improve the separation of SILMs, has been deeply studied for metallic nanoparticles as well. For example, the addition of Cu nanoparticles to ILs caused their surface to become positively polarized and resulted in increased CO_2_ permeance and selectivity [72]. Different ILs, such as [HMIM][NO_3_], [OMIM][BF_4_] or [BMIM][NO_3_], have been tested with PES supports [73,74] and [BMIM][BF_4_] with KIT-6 silica support [75]. The factors that affect the performance of this type of SILMs with Cu nanoparticles have been deeply investigated [76].

Another very innovative option for improvement in CO_2_ capture is the consideration of processes based on biological systems, mainly taking into account the employment of carbonic anhydrase enzymes, which catalyse the reversible hydration of CO_2_ to produce bicarbonate. The first proposed approach to implement this enzymatic technology was the Carbozyme process [77]. It is based on the contact between CO_2_ in flue gas streams and carbonic anhydrase aqueous solution. The produced bicarbonate diffuses across a liquid membrane to get converted back to CO_2_ upon desorption in the presence of vacuum or a sweep gas in the permeate side. The main problem with this technology is the water management, as water evaporates (even at relatively low temperatures). Nevertheless, in order to prevent liquid loss, ILs were considered to replace water as solvent and the implementation of SILMs emerged as an intensification option for the Carbozyme process thanks to the combined effect between the increased uptake of CO_2_ by the IL and the additional enzymatic conversion mechanism. The research group headed by Prof. J. G. Crespo proposed [BMIM][NTf_2_] with carbonic anhydrase in hydrophobic PVDF supports for this purpose [78]. The obtained results demonstrated that the SILMs were stable at high temperatures and selective towards CO_2_ when compared with N_2_. Moreover, the presence of the enzyme increased the CO_2_ solubility coefficient up to 30%, even if a low enzyme concentration was employed. Further research has been carried out to improve the performance of these enzymatically-boosted SILMs. For example, the avoidance of highly-purified, commercially available carbonic anhydrase, which is extremely expensive since blood is mainly used as its source, has been investigated. Alternative routes have already demonstrated the recovery of this enzyme from biomass. This recovered enzyme has been applied to the design of SILMs totally equivalent to those previously applied by the group of Prof. Crespo to CO_2_ separation [79]. The results indicate that the SILMs with the enzyme derived from spinach also possessed the increased ability to permeate CO_2_, as demonstrated when comparing the results with enzymeless controls. More recent research has been focused on the improvement of the thermal stability of these enzymatic SILMs [80]. Once again, PVDF membranes were impregnated with [BMIM][NTf_2_] but in this case they contained carbonic anhydrase, which had been obtained from the thermophilic bacterium *Sulfurihydrogenibium yellowstonense*. The results obtained showed that the SILMs prepared present interesting permeability at higher temperatures (up to 100 °C) and preserve good transport selectivity towards CO_2_ against N_2_.

Nevertheless, although the advances in the study of SILMs for CO_2_ separation have been great, there is still a need for further improvement. On the one hand, SILMs are rarely tested under real industrial conditions. More realistic conditions must be selected to characterize the performance of the proposed SILMs at high pressures, high temperatures and different gas mixture compositions. On the other hand, improved chemical and mechanical stability during long-term tests is required to demonstrate the potential of SILMs in a real industrial-scale implementation [1]. In addition to these two main concerns, other relevant aspects that deserve further attention have been identified, such as a better understanding of the structure-related properties of ILs and their interactions with the support materials or the development of improved separations process by means of advanced membrane/IL configurations, like PIMILs and GILMs [64].

#### 3.3.2. Other Gas Separations

The employment of SILMs has demonstrated its convenience to enhance gas separation performance by improving both permeability and selectivity for several industrially-relevant gas mixtures apart from CO_2_ mixtures [81]. SILMs have been investigated for the absorption or separation of sulphur gases [82]. Five imidazolium-based ILs supported on PES microfiltration membranes were studied for SO_2_ separation. The tested SILMs exhibited very good permeability of SO_2_ and high selectivity when mixed with CH_4_ or N_2_, with values an order of magnitude higher than those of CO_2_ [83]. PVDF membranes were also investigated to support [MIM][Ac] and [BIM][Ac] and demonstrated their potential to selective remove SO_2_ from air. With only SO_2_ 10% (in volume) in the air feed, the total permeability through the membrane increased more than 5 times comparing with the pure air permeability [84]. SILMs for separation of acidic gases, including SH_2_, from crude natural gas have been prepared using PVDF as polymeric matrix and [BMIM][BF_4_] as IL [85]. Values above 150 were achieved for SH_2_/CH_4_ selectivity, as well as high permeation and good mechanical stability at high operating pressure. Moreover, Seeberger and co-workers carried out work to analyse all the possibilities for desulfurization of gaseous mixtures by SILMs [86]. As a consequence of the satisfactory results obtained, they proposed [BMIM][NTf_2_] in expanded PTFE or in alternative hydrophilic polymeric membranes for SH_2_ separation from biogas, SO_2_ separation from flue gas and removal of tetrahydrothiopene (a sulphur-based natural gas odorant) from natural gas.

Other gas mixtures that have been studied for SILMs application must be highlighted, such as nitrogen-enriched mixtures or propane/propylene mixtures. On the one hand, H_2_, O_2_ and CO have been removed by SILMs from mixtures with N_2_. The separation of hydrogen from the inert nitrogen-enriched atmosphere employed in fermentators was performed by imidazolium based ILs in PVDF support [87]. Although the selectivity values for CO_2_/N_2_ were higher than those for H_2_/N_2_, this last separation was possible. Other authors found that [MIM][Cl] and its mixtures with CuCl immobilized in a hydrophilic PVDF membrane could be useful for the simultaneous recovery of H_2_ and CO from N_2_ enriched streams due to the combination of a high H_2_ permeability and an improved CO permeability over that of N_2_ [88]. This same research group had previously investigated the facilitated transport of CO over N_2_ through this type of SILMs and the effects of different operating conditions, such as pressure, temperature, sweep gas flowrate and liquid membrane composition [89]. The separation of O_2_ from N_2_ in air is an important process that is widely applied in industry and efforts to include SILMs as an alternative technique have been identified [90]. Porous alumina tubes were impregnated with [EMIM][BF_4_] and, although the maximum O_2_/N_2_ selectivity was around 6, the SILM was much more stable than membranes supporting a more selective carrier (perfluorotributylamine). Additional examples of the application of SILMs to separate other gases from N_2_ atmosphere have been found, such as the removal of dioxins from high temperature gas [91]. Two different ILs, [Aliquat][DCA] and [OMIM][DCA], inside the porous structure of ceramic (titania or alumina) membranes were proposed for this purpose. Experiments with model incineration gas were also carried out and the obtained results validated the option of using these SILMs for the removal of dioxins from high temperature sources. On the other hand, the similar boiling points of propane and propylene difficult the separation of these compounds obtained from catalytic naphtha cracking and complex and expensive separation equipment is required. Consequently, alternative options to distillation have been investigated and SILMs have been proposed. The separation of a propylene/propane vapour mixture can be enhanced by selective conversion of propylene to hexenes via a homogeneously catalysed dimerization reaction in a SILM [92]. The most promising combination was [BMIM][NTf_2_] in an asymmetric ceramic support, which displayed sufficient permeability, reasonable selectivity and long-term stability. Other SILMs were used for propane/propylene gas mixtures separation using AgBF_4_ dissolved in [BMIM][BF_4_] as carrier solution with hydrophilic PVDF polymeric supports [93]. The effects of operation variables such as the transmembrane pressure, sweep gas flow rate and silver concentration on the separation process were investigated. Unfortunately, these SILMs loosed efficiency at operating times longer than 90 min because the silver ions were reduced to metallic silver. 

#### 3.3.3. Pervaporation

Pervaporation is an effective combination of membrane permeation and evaporation, which appears as an attractive alternative to other separation methods for liquid mixtures [94]. The liquid feed mixture is in direct contact with the active surface of the membrane and the permeate comes out as vapour from the opposite side of the membrane, which is kept under vacuum or continuous gas sweeping. This continuous removal of the vaporous permeate creates a concentration gradient across both sides of the membrane. Some examples of implementation of SILMs to pervaporative separations have been published. The most common applications of pervaporation based SILMs are organic-organic mixture separation, dehydration of organic-water mixtures and removal of volatile organic compounds from water [95,96].

The search for new routes for the synthesis of biofuels has promoted the microbial production of bio-butanol. The bacterium *Clostridium acetobutylicum* is able to produce acetone butanol and ethanol by way of the process known as clostridial acetone-butanol-ethanol (ABE) fermentation [97]. The high separation costs associated with the very low concentrations of the fermentation products and the toxicity of butanol over the microorganisms require effective separation techniques to avoid limited productivity. Pervaporation appears as an advantageous alternative over distillation and SILMs have been investigated for this application in order to remove bio-butanol from aqueous solutions or from fermentation broths. Izak and co-workers demonstrated the improved separation properties of [EeMIM][PF_6_] and [TPA][TCB] with PDMS over ceramic ultrafiltration membranes when compared to membranes without ILs, for the removal of butanol and acetone from fermentation culture broth [98,99]. Other ILs based on ammonium and phosphonium have been successfully applied to recover butanol from dilute aqueous solutions. The dilution of these ILs with oleyl alcohol enhanced separation performance but only temporarily, since the alcohol was gradually leached during the experiments [100]. Improvements in the stability of SILMs have been a major concern and different options have been investigated [101]. The immobilization by inclusion in a polyether block amide (PEBA) polymer matrix of ILs such as [DMIM][FAP], [DMIM][TCB] and [TTHB][TCB] [102] or [HMIM][TCB] [103] resulted in extended effective lifetime of the SILMs. Nevertheless, in some cases an additional silicone coating was necessary to achieve the stable immobilization, which resulted in decreased permeability. An alternative option is the employment of gelled ILs, like in the case of gelled [BMIM][PF_6_] in PTFE for the separation of ABE mixtures from aqueous solutions [104]. Whole the permeability of the SILM resulted comparable to a membrane evaporation system, the system was much more selective to the butanol transfer and increased the butanol/ethanol selectivity.

Other alcohols and water mixtures have been object of SILM pervaporation. For instance, 1–3 propanediol aqueous solutions were treated with [TPA][TCB] in ceramic nanofitration membrane and the impregnation of the support increased the selectivity but decreased the permeability [105]. Moreover, the performance of [BMIM][BF_4_], [BMIM][PF_6_] and [HMIM][PF_6_] in porous Matrimid membranes was evaluated to investigate their potential for water and organic mixtures [106]. The results identified [BMIM][BF_4_] as the most promising IL to obtain SILMs able to separate cyclohexane from water or ethanol. Pervaporation with SILMs can be especially recommended for the separation of azeotropic mixtures. A clear example is the recovery of ethyl acetate, an important solvent difficult to be recovered from diluted aqueous solutions because it forms an azeotrope. The recovery of ethyl acetate using [BMIM][BF_4_] embedded in the P(VDF-HFP) matrix resulted successful [107]. Even a ternary azeotrope including ethanol in the ethyl-acetate mixture was effectively treated with the same IL but in this case blended with polyvinyl alcohol (PVA) and supported in buckypaper [108]. This SILM had been previously investigated to be applied to the pervaporative dehydration of ethylene glycol [109].

#### 3.3.4. Separations in Liquid Phase

The most deeply studied application of SILMs to liquid phase separations is the selective separation of organic compounds, with the first examples published among the earliest documents related to SILMs [110,111]. The separation of aromatic and paraffin hydrocarbons has been widely investigated. As observed in Table 6, which summarizes the cases of SILMs applied to hydrocarbon separations, the investigated paraffins include aliphatic and cyclic hydrocarbons, while all the members of the BTEX family (Benzene, Toluene, Ethylbenzene and Xilenes) are included among the investigated aromatic compounds. Since the initial studies that demonstrated that these aromatic hydrocarbons were successfully transported through SILMs based on ILs such as [BMIM][PF_6_], [HMIM][PF_6_], [OMIM][PF_6_] or [Et2MeMoEtN][NTf_2_] [112], new research efforts were focused on the improvement of the selectivity of the SILMs and their stability [113,114,115,116,117]. Moreover, SILMs have demonstrated their validity for the removal of aromatics compounds from aqueous phases. The transport of phenol through SILMs from aqueous feed solutions to NaOH stripping solutions was investigated for PVDF [118] and PTFE [119] supports. While 74% of the phenol present in the feed phase was transported to the stripping phase within 24 h using CYPHOS 104 in PVDF, up to 85% was removed by using [BMIM][HSO_4_] in PTFE. The removal of bisphenol A from aqueous solutions was not so effective [120]. The maximum value of permeation achieved with [TBP][PF_6_] in PVDF did not attain 63%, even when feed pH control was implemented. Examples of the potential of SILMs to remove other organic pollutants from wastewaters have been identified. An SILM based on [BDMIM][PF_6_] in PVDF was successfully applied to the removal of the pesticide endosulphan from wastewater [121]. Even the selective transport of a more complex aromatic compound like lignin through SILMs has been investigated [122].

As a consequence of the increasing demand for environmentally friendly processes, fermentation has become a very important route to obtain organic compounds [123]. The economic viability of fermentators requires the development of effective separation methods for optimal broth preparation and recovery and purification of the required products. In situ, extractive fermentation with SILMs for the recovery of organic acids has been investigated. Matsumoto and co-workers have deeply analysed the permeation of lactic and succinic acids through SILMs [124,125]. Lactate was successfully permeated through PVDF membranes impregnated with Aliquat 336, CYPHOS 101 and CYPHOS 102 but the higher stability of PIMILs based on the same ILs but in PVC membranes was demonstrated [126]. Aliquat 336 in PVDF support was a useful SILM for the selective transport of succinic acid as well and with HCl as receiving phase (pH < 2) it can occur even against gradient [127]. The same research group has also been focused on the applicability of SILMs to the selective transport of saccharides (mainly hexoses, pentoses and disaccharides). They concluded that the saccharides successfully permeated through SILMs based on Aliquat 336 in PVDF support [128] but selective separations were achieved when carriers were incorporated into the SILM [129]. Whereas high facilitated transport of glucose occurred with calix [4] arene and calix [6] arene as carriers, calix [8] arene was more effective for the selective transport of fructose, arabinose or ribose.

The application of SILMs to separate transesterification reaction products and substrates has been investigated. The most analysed case is the separation of vinyl butyrate, 1-butanol butyl butyrate and butyric acid [130,131,132]. The evaluation of five different polymeric membranes to support [BMIM][PF_6_] was carried out and Nylon resulted the best alternative. Therefore, other ILs were immobilized in that support to study the influence of the IL on the selective separation of the compounds. Another example of SILMs applied to separate transesterification compounds is the kinetic resolution of rac-1-phenylethanol using a membrane bioreactor containing a SILM based on [BMIM][BF_4_] in Nylon [133]. This research demonstrated that the coupling of the lipase enantioselectivity with the selective separation of SILMs provides a promising basis for practical production of enantiomerically pure or enriched compounds.

The potential of SILMs to improve the desulfurization of crude oil and derived fuels has been analysed as well. The feasibility of using SILMs for the desulfurization of hydrocarbon streams, such as raw diesel and whole crude oil, was investigated [134]. The studied SILMs ([BMP][MeSO_4_] in PES, PVDF and PTFE) exhibited very low mass transfer coefficients, as the formation of fouling material on the membrane surface slowed the transport. For the case of the desulfurization of jet fuels, a mixture of n-dodecane and 1-hexanethiol was selected and three imidazolium based ILs were tested in different polymeric supports [135]. The permeation of n-dodecane observed through the SILMs was noteworthy and an alternative design with the IL as a receiving phase in the downstream side of a hollow fibre membrane contactor was suggested. Moreover, organic nitrogen compounds (such as quinolone, indole, pyridine and carbazole) inhibit sulphur removal by hydrodesulphurisation. A SILM based on Zn-containing [EMIM][EtSO_4_] in PVDF resulted a satisfactory option to selective remove these undesired compounds from n-heptane as model compound [136].

Apart from organic compounds, another interesting field of application of SILMs is the removal of metal ions from aqueous solutions. During last years, several studies have deal with the permeation of a number of metals through SILMs using different membrane configurations and ILs. Cyphos IL101, supported in PVDF, has been tested for selective gold transport [137]. The system resulted reasonably selective against the presence of base metals, such as Ni(II), Cu(II) and Fe(III), in the feed solution. Three members of the Cyphos family (IL 101, IL 104 and IL 168) were employed to compare the transport of Zn(II), Fe(II) and Fe(III) through SILMs and PIMILs based on PVDF and cellulose triacetate respectively [138]. On one hand, separation of Zn(II) and Fe(III) from Fe(II) was more effective with PIMILs but, on the other hand, SILMs resulted more stable in consecutive steps of extraction-washing cycles. The transport of Cr(VI) using once again CYPHOS IL101 supported in PVDF was satisfactorily accomplished and optimized [139]. The optimal conditions regarding the compositions of the feed and stripping phase were identified. The influence of the stripping phase composition has been investigated by other authors, since the selective separation of different target metallic ions can be optimized just by selecting the most appropriate stripping phase [140]. SILMs based on [MTOA][Cl] in Nylon were used for the selective extraction of Fe(III), Zn(II), Cd(II) and Cu(II). While the selection of a NH_3_ (6 M) solution allowed the recovery of Cd(II), Fe(III) was recovered using ultrapure water as stripping phase and Na_2_CO_3_ (0.1 M) promoted the recovery of Zn(II). Vanadium was another effectively transported metal, in this case, employing a PTFE support impregnated with [MTOA][Cl] [141]. Moreover, it was observed that adding a slight amount of a second IL ([BMIM][NTf_2_]) improved the V(IV) extraction process remarkably. The selective recovery of rare earth metals by [OMIM][NTf_2_] supported in PVDF was investigated [142]. Quantitative transport of Dy and Nd through the SILM occurred, while Fe was only slightly transferred, so the applicability of the system to the recovery of these valued metals from the leaching solution of magnet scrap was proposed.

#### 3.3.5. Other Relevant Applications

The application of SILMs in analytical chemistry is a very relevant research issue. Before SILMs were applied in this field, SLMs based on organic solvents had been successfully employed to separate and concentrate diverse complex matrices for analytical purposes due to their extraordinary selectivity, easy accessibility, simplicity, high pre-concentration ability and excellent sensitivity [12,143]. However, the typical drawbacks caused by the evaporation or dissolution of the organic solvents have promoted the replacement of these organic solvents by ILs to investigate the potential of SILMs. The unique physicochemical properties of ILs make SILMs very promising candidates as extraction media for a range of microextraction techniques. Since ILs were used for first time in liquid-phase microextraction [144], their application has become widespread in the extraction tasks within the analytical field [145]. The combination of these ILs with membranes, mainly hollow fibres, was the basis of the implementation of SILMs for sample preparation before analysis [146].

Organic emerging pollutants are becoming a great concern, as their presence in environmental waters is a threat to human health and aquatic ecosystems. The determination of these compounds in aqueous sample is critical and, consequently, the development of reliable, sensitive, inexpensive and easily operating detection methods must be promoted. Sample preparation and clean-up in order to eliminate the interference of matrices for environmental samples is of great importance and SILMs have a great role to play. The analytes can be extracted from aqueous samples, through a thin layer of IL (just several microlitres) immobilized within the pores of a porous hollow fibre and transferred to an acceptor phase inside the lumen of the hollow fibre [147]. This technique can provide a high analyte preconcentration and excellent sample clean-up, with the advantage that the hollow fibre can be disposed after use because of its low cost. The identification of an article about the direct determination of chlorophenols as pollutant in environmental water samples using [OMIM][PF_6_] immobilized in PP [50] among the most cited documents in Table 5 gives a clear idea about the relevance of the research work in this application. Even new efforts continue focusing on the improvement of the determination of these specific compounds by incorporation of a more hydrophobic IL such as [HMIM][FAP] [148]. Pharmaceuticals are other important analytes that have been subject of attention and documents related to their analytical determination supported by SILMs have been found, which cover antibiotics such as sulfonamides [149] or kanamycin [147] and antidepressants [150], in this last case in an electrically assisted process. The investigation of the relative parameters that affect extraction efficiency and selectivity (such as type of ionic liquid membrane, presence of a carrier, extraction time, pH of the donor sample and acceptor phase, stirring conditions during extraction, or sample salinity) is the main objective of the researchers in this topic, in order to optimize the conditions and propose the most convenient analytical methods. Simple hollow fibre supported liquid phase microextraction (HF–LPME) with ILs can also be applied to the determination of metal concentrations in environmental samples, with examples of their employment for hexavalent chromium [151] and mercury [152], which use polypropylene supports with Aliquat 336 and [BMIM][PF_6_] with 0.02% dithizone as ILs, respectively.

The potential use of SILMs within fuel cells is another very hot topic. The fuel cells are devices that employ a kind of chemical fuel as a source of energy [153]. Among all the available options, proton exchange membrane fuel cells (PEMFCs) are one of the most promising solutions for automotive and stationary power sources, since hydrogen is used as fuel and only water is obtained as by-product. The proton exchange membrane (PEM) is a crucial component of this type of cells, as it is required to ensure a good chemical, mechanical and electrochemical stability. Besides its role in physically separating the two half-cells, it is supposed to facilitate the selective transport of protons from the anode to the cathode. Currently, perfluorinated sulfonic acid membranes satisfy these specifications and Nafion must be considered as the reference material. However, some drawbacks, like its high cost, the complicated water management and the low power density at temperature above 100 °C, restrict the use of Nafion [154]. ILs have been considered as potential additives for PEMs due to the interesting properties (such as high proton conductivity and high solvent power) that make them promising anhydrous proton carriers. The performance of Nafion and alternative membranes can be improved due to their impregnation, intercalation or reaction with ILs [155]. Apart from impregnation to obtain SILMs, ILs can be incorporated into PEMs through different methods: direct addition of the IL into casting solution, anchoring of the IL on a filler followed by incorporation into the polymer matrix or direct grafting the IL on the polymer [156]. For further information, critical reviews of the progress in the application of ILs to PEMs for fuel cells are available [157,158].

Examples of SILMs applied to get improved PEMs have been identified. The impregnation of polyimide membranes with imidazolium ILs, like [MIM][DBP], [BIM][DBP] and [BIM][BEHP], resulted in conductivity values sufficiently high compared to Nafion when tested at temperatures above 80 °C [159]. Other polymeric membranes made of PVDF-hexafluoropropene and Nafion were employed as supports for [BMIM][OTf] and [EIM][OTf] [160]. The prepared SILMs showed satisfactory ionic conductivity but temperatures above 100 °C resulted in a low output performance of the fuel cell. 

Direct methanol fuel cells (DMFCs) can also take advantage of the use of SILMs. Imidazolium ILs with different anions, cations, in different concentrations and incorporated using different solvents have been analysed to determine the effects of all these variables in the chemical, thermal, morphological and transport properties of membranes made of sulfonated poly(styrene-isobutylene-styrene). The results demonstrated that there is an optimum amount of IL that simultaneously allows enough water for efficient proton conductivity and minimum methanol permeability in a DMFC [161].

Microbial fuel cells (MFCs) converts chemical energy to electrical energy by the action of microorganisms. Bacteria oxidize organic matter and transfer the resulting electrons directly to an electrode (or to a redox mediator). When the organic matter present in wastewaters is employed as fuel, MFCs can simultaneously produce energy and purify wastewater. As in other fuel cells, the performance of PEMs clearly determines the efficiency of MFC. Research aimed to evaluate the potential use of SILMs as PEMs in MFCs has been found [162]. The tested SILMs ([MTOA][Cl] in Nylon support being the best example) achieved power and chemical oxygen demand removal similar to, or even higher than, conventional proton exchange membranes such as Nafion or Ultrex. Other SILMs, which used PVDF membranes to support [BMIM][NTf_2_]) and [HMIM][PF_6_], were tested and the results were compared to MFCs operating with Nafion [163]. The experiments shown that the SILMs could be competitive with the Nafion at low substrate inputs but further investigation is required to correlate the ionic liquid properties with the MFC performance. PIMILs are produced by mixing relatively inert polymers with ionic liquids during the casting tasks. Recently, it has been demonstrated the higher stability of PIMILs compared with SILMs and their potential use as PEMs has been studied [164]. The higher amount of immobilized [MTOA][Cl] in PVC when compared to the same IL supported in Nylon involved an increase of the MFC power. These results suggested that PIMILs s might be preferred over SILMs or conventional PEMs to be incorporated in MFCs.

## 4. Conclusions

This study carried out a complete overview of the research on supported ionic liquid membranes (SILMs) during the 1995–2015 period, with the information related to annual publications, document types, languages, subjects, journals, countries and institutions. Further analysis identified the most relevant applications and the corresponding research emphases and trends. The earliest document found was published in 1998 and the production until 2005 was very limited. Although *Chemical Engineering* was the dominant subject, *Chemistry* appeared very close, fact that gives an idea about the collaborative work carried out between chemical engineers and chemists in this field. The journal that published more documents was *Journal of Membrane Science* but other two journals highly related to separation processes completed the podium. While USA was the most productive country, just followed by China, the third position attained by Spain must be highlighted, since 3 Spanish universities were identified among the top 5 most productive institutions.

The analysis of keywords provided the clues for the identification of the most important applications of SILMs. The separation of CO_2_ was clearly the most relevant application and much research effort has been focused on this topic. The potential of nanotechnology and the integration of a biomolecular resource such as enzymes must be highlighted among the hottest trends for CO_2_ separation. Other gas phase separations that attract much scientific interest include the removal of sulphur gases and the processing of nitrogen-enriched or propane/propylene mixtures. Pervaporation is another topic where SILMs have demonstrated their usefulness, mainly applied to the production of bio-butanol by ABE fermentation. Liquid phase separations have been investigated to less extension than gas phase separations. Nonetheless, the separation of a great diversity of organic compounds has been proposed but the selective transport of metallic ions should be mentioned as well. SILMs have been successfully applied to analytical purposes and a very recent and hot trend that has been identified is their potential use within fuel cells.

## Figures and Tables

**Table 1:** The top 6 most popular subject categories.

Ranking	Subject Categories	Documents	Percentage (%)
1	*Chemical Engineering*	135	72.2
2	*Chemistry*	125	66.8
3	*Materials Science*	61	32.6
4	*Engineering*	43	23.0
5	*Biochemistry, Genetics and Molecular Biology*	42	22.5
6	*Environmental Science*	29	15.5

**Table 2:** The top 5 most productive journals.

Ranking	Journal	IF (WoS)	SJR (Scopus)	Documents	Percentage (%)
1	*Journal of Membrane Science*	5.557	2.000	35	20.8
2	*Separation and Purification Technology*	3.299	1.078	14	8.3
3	*Desalination*	4.412	1.522	8	4.8
4	*Chemical Engineering Journal*	5.310	1.695	6	3.6
5	*Industrial and Engineering Chemistry Research*	2.567	0.949	6	3.6

**Table 3:** The top 10 most productive countries.

Country	TP	(%)	SPR	(%)	ICPR	(%)	FAPR	(%)	CAPR	(%)
USA	42	(22.5)	1	(23.4)	2	(19.4)	1	(21.6)	1	(21.6)
China	33	(17.8)	2	(20.1)	10	(6.5)	2	(17.8)	2	(17.8)
Spain	26	(14.1)	3	(14.9)	7	(9.7)	3	(13.0)	3	(13.0)
Japan	16	(8.6)	4	(7.1)	3	(16.1)	4	(7.0)	4	(7.0)
Germany	14	(7.6)	6	(4.6)	1	(10.3)	6	(4.9)	6	(4.9)
Portugal	14	(7.6)	5	(5.8)	3	(16.1)	4	(7.0)	4	(7.0)
Czech Republic	7	(3.8)	13	(1.3)	3	(16.1)	8	(2.7)	8	(2.7)
India	7	(3.8)	7	(3.2)	10	(6.5)	8	(2.7)	8	(2.7)
Poland	7	(3.8)	7	(3.2)	10	(6.5)	7	(3.8)	7	(3.8)
UK	6	(3.2)	9	(1.9)	7	(9.7)	8	(2.7)	8	(2.7)

TP: Total publications; SPR: Single country publication rank; ICPR: International collaboration publication rank; FAPR: First author publication rank; CAPR: Corresponding author publication rank.

**Table 4:** The top 8 most productive institutions.

Ranking	Institutions	Documents	Percentage (%)
1	Polytechnic University of Cartagena (Spain)	14	7.5
2	National Energy Technology Laboratory, Morgantown (USA)	13	7.0
3	Doshisha University (Japan)	11	5.9
4	University of Murcia (Spain)	10	5.3
5	University of Cantabria (Spain)	8	4.3
6	Institute of Chemical Process of the Academy of Sciences (Czech Republic)	7	3.7
7	Centre for Fine Chemistry and Biotechnology (Portugal)	7	3.7
8	Oak Ridge National Laboratory (USA)	6	3.2

**Table 5:** The top 10 most cited papers.

Ranking	Articles	Times Cited
1	Title: Guide to CO_2_ separations in imidazolium-based room-temperature ionic liquids Author(s): Bara, J.E.; Carlisle, T.K.; Gabriel, C.J.; Camper, D.; Finotello, A.; et al. Source: *Industrial and Engineering Chemistry Research* Published: 2009	409
2	Title: Gas separations using non-hexafluorophosphate [PF_6_]^−^ anion supported ionic liquid membranes Author(s): Scovazzo, P.; Kieft, J.; Finan, D.A.; Koval, C.; DuBois, D.; Noble, R. Source: *Journal of Membrane Science* Published: 2004	298
3	Title: State-of-the-art of CO_2_ capture with ionic liquids Author(s): Ramdin, M.; De Loos, T.W.; Vlugt, T.J.H. Source: *Industrial and Engineering Chemistry Research* Published: 2012	259
4	Title: Ionic liquids for CO_2_ capture—Development and progress Author(s): Hasib-ur-Rahman, M.; Siaj, M.; Larachi, F. Source: *Chemical Engineering and Processing: Process Intensification* Published: 2010	244
5	Title: Direct determination of chlorophenols in environmental water samples by hollow fibre supported ionic liquid membrane extraction coupled with high-performance liquid chromatography Author(s): Peng, J.F.; Liu, J.F.; Hu, X.L.; Jiang, G.B. Source: *Journal of Chromatography A* Published: 2007	195
6	Title: Examination of the potential of ionic liquids for gas separations Author(s): Baltus, R.E.; Counce, R.M.; Culbertson, B.H.; Luo, H.; et al. Source: *Separation Science and Technology* Published: 2005	162
7	Title: Determination of the upper limits, benchmarks and critical properties for gas separations using stabilized room temperature ionic liquid membranes (SILMs) for the purpose of guiding future research Author: Scovazzo, P. Source: *Journal of Membrane Science* Published: 2009	160
8	Title: Recent advances in supported ionic liquid membrane technology Author(s): Lozano, L.J.; Godínez, C.; de los Ríos, A.P.; et al. Source: *Journal of Membrane Science* Published: 2011	136
9	Title: SO_2_ gas separation using supported ionic liquid membranes Author(s): Jiang, Y.Y.; Zhou, Z.; Jiao, Z.; Li, L.; Wu, Y.T.; Zhang, Z.B. Source: *Journal of Physical Chemistry B* Published: 2007	132
10	Title: High temperature separation of carbon dioxide/hydrogen mixtures using facilitated supported ionic liquid membranes Author(s): Myers, C.; Pennline, H.; Luebke, D.; et al. Source: *Journal of Membrane Science* Published: 2008	128

**Table 6:** Examples of use of SILMs in hydrocarbon separations.

Reference	Aromatic HC	Paraffin HC	IL	Support
Zhang 2015	Toluene	*n*-heptane	[BMIM][BF_4_]	PVDF
Zhang 2014	Toluene	Cyclohexane	[BPy][BF_4_]	PVDF
	Toluene	Cyclohexane	[BMIM][BF_4_]	PVDF
Chakraborty 2012	Benzene	*n*-heptane	[OMIM][Cl]	PP
	Toluene	*n*-heptane	[OMIM][Cl]	PP
	Ethylbenzene	*n*-heptane	[OMIM][Cl]	PP
	*p*-xylene	*n*-heptane	[OMIM][Cl]	PP
	Benzene	*n*-heptane	[EMIM][EtSO_4_]	PP
	Toluene	*n*-heptane	[EMIM][EtSO_4_]	PP
	Ethylbenzene	*n*-heptane	[EMIM][EtSO_4_]	PP
	*p*-xylene	*n*-heptane	[EMIM][EtSO_4_]	PP
	Benzene	*n*-heptane	[MIM][HSO_4_]	PP
	Toluene	*n*-heptane	[MIM][HSO_4_]	PP
	Ethylbenzene	*n*-heptane	[MIM][HSO_4_]	PP
	*p*-xylene	*n*-heptane	[MIM][HSO_4_]	PP
Zeng 2008	Benzene	Cyclohexane	[BMIM][PF_6_]	Not identified
Matsumoto 2006	Benzene	Cyclohexane	[Et_2_MoEtN][BF_4_]	PES
Matsumoto 2005	Benzene	*n*-heptane	[BMIM][PF_6_]	PVDF
	Toluene	*n*-heptane	[BMIM][PF_6_]	PVDF
	*p*-xylene	*n*-heptane	[BMIM][PF_6_]	PVDF
	Benzene	*n*-heptane	[HMIM][PF_6_]	PVDF
	Toluene	*n*-heptane	[HMIM][PF_6_]	PVDF
	*p*-xylene	*n*-heptane	[HMIM][PF_6_]	PVDF
	Benzene	*n*-heptane	[OMIM][PF_6_]	PVDF
	Toluene	*n*-heptane	[OMIM][PF_6_]	PVDF
	*p*-xylene	*n*-heptane	[OMIM][PF_6_]	PVDF
	Benzene	*n*-heptane	[Et_2_MeMoEtN][NTf_2_]	PVDF

## Nomenclature ILs

**Symbol**	**Name**
[BEHP]	Bis(2-ethyl-hexyl)-phthalate
[BF_4_]	Tetrafluoroborate
[BIM]	Butyl-imidazolium
[BMIM]	Butyl-methyl-imidazolium
[BMP]	Butyl-methyl-pyridinium
[BPy]	*N*-butyl-pyridinium
[Cl]	Chloride
[DBP]	Di-butyl-phosphate
[DCA]	Dicyanoamide
[DMIM]	Decyl-methyl-imidazolium
[EIM]	Ethyl-imidazolium
[EeMIM]	Ethenyl-methyl-imidazolium
[Et_2_MeMoEtN]	Di-ethyl-methyl-(2-methoxyethyl)-ammonium
[Et_2_MoEtN]	Di-ethyl-(2-methoxyethyl)-ammonium
[EtSO_4_]	Ethylsulfate
[FAP]	Tris(pentafluoroethyl)-trifluorophosphate
[HMIM]	Hexyl-methyl-imidazolium
[HSO_4_]	Hydrogensulfate
[MeSO_4_]	ylsulfate
[MIM]	Methyl-imidazolium
[MTOA]	Methyl-trioctyl-ammonium
[NO_3_]	Nitrate
[NTf_2_]	Bis((trifluoromethyl)sulfonyl)-imide
[OMIM]	Octyl-methyl-imidazolium
[OTf]	Triflate
[PF_6_]	Hexafluorophosphate
[TBP]	Tetrabutyl-phosphonium
[TCB]	Tetracyanoborate
[TPA]	Tetrapropyl-ammonium
[TTHB]	Tetradecyl-trihexyl-phosphonium

## Nomenclature Polymers

**Acronym**	**Name**
PDMS	Polydimethylsiloxane
PEBA	Polyether block amide
PES	Polyethersulfone
PP	Polypropylene
PTFE	Polytetrafluoroethylene
PVA	Polyvinyl acetate
PVDF	Polyvinylidene fluoride
P(VDF-HFP)	Poly(vinylidene fluoride-co-hexafluoropropylene)
